# 

**Published:** 1884-05

**Authors:** 


### Contents—May.

ORIGINAL COMMUNICATIONS:

Fermentation in the Human Mouth. W. D. Miller.................. 225
Dental Mechanism. A. Retter........................................ 233
Dental Hygiene. S. B. Palmer....................................... 237
The D. D. S. in Europe. J. L. Tierney.............................. 241
Capillary Dentures. C. H. Land..................................... 243
The Wisconsin Dental College. Adolf Pettermann..................... 244
Is Dental Work Expensive. D. R. Stubblefield....................... 250
Incident of Practice. C. E. Francis................................ 253

REPORTS OF SOCIETY MEETINGS:

Mississippi Valley Dental Association.............................. 254
New York Odontological Society..................................... 258
Fifth District Dental Society of the State of New York............. 261

EDITORIAL:

Filling New Canals................................................. 262
Toothache Remedy................................................... 266
Bogus Diplomas..................................................... 266
Our Book Table..................................................... 267

CURRENT NEWS AND OPINION:

"Too Busy"......................................................... 271
The Therapeutic Application of Nitrous Oxide Gas................... 272
Illinois State Dental Society...................................... 273
Kansas State Dental Society........................................ 274
Dental Meetings for May............................................ 275
Dental Society of the State of New York............................ 275
Perpetual Injunction............................................... 276
American Medical Association....................................... 276
A Case of Œsophagotomy............................................. 276
Michigan Dental Association........................................ 277
Sixth District Dental Society of New York.......................... 277
Cicero on Dentistry................................................ 277
Personal........................................................... 278
Second District Dental Society..................................... 278
Mad River Valley Dental Society.................................... 278
Northern Ohio Dental Association................................... 279
Southern Dental Association........................................ 279
Connecticut Valley Dental Association.............................. 279
Askings and Answers................................................ 280

